# RETRACTION: Establishment and Verification of a Method for Analyzing Nasal Blackheads Images

**DOI:** 10.1111/srt.70221

**Published:** 2025-08-25

Sun H, Yang GS, Yuan J, Jiang Y, Jin G. Establishment and verification of a method for analyzing nasal blackheads images. *Skin Res Technol*. 2024;30(3):e13648. doi:10.1111/srt.13648

The above article, published online on 13 March 2024 in Wiley Online Library (wileyonlinelibrary.com), has been retracted by agreement between *Skin Research and Technology*; and John Wiley & Sons Ltd. The article was accepted for publication following an insufficient peer review process that does not meet the standards of the journal's peer review policy. Furthermore, the article fails to provide essential information necessary for interpreting and reproducing the findings, and lacks adequate support from the existing literature. Accordingly, the article has been retracted. The authors have been informed of this decision and disagree with it.

